# Exploring BOLD Changes during Spatial Attention in Non-Stimulated Visual Cortex

**DOI:** 10.1371/journal.pone.0005560

**Published:** 2009-05-15

**Authors:** Linda Heinemann, Andreas Kleinschmidt, Notger G. Müller

**Affiliations:** 1 Cognitive Neurology Unit & Brain Imaging Center, Clinic for Neurology, Johann Wolfgang Goethe-University, Frankfurt, Germany; 2 Institute for Medical Psychology, Johann Wolfgang Goethe-University, Frankfurt, Germany; 3 INSERM, Unité 562, Gif-sur-Yvette, France; 4 Department of Neurology, Otto-von-Guericke-University, Magdeburg, Germany; University of California Davis, United States of America

## Abstract

Blood oxygen level-dependent (BOLD) responses were measured in parts of primary visual cortex that represented unstimulated visual field regions at different distances from a stimulated central target location. The composition of the visual scene varied by the presence or absence of additional peripheral distracter stimuli. Bottom-up effects were assessed by comparing peripheral activity during central stimulation vs. no stimulation. Top-down effects were assessed by comparing active vs. passive conditions. In passive conditions subjects simply watched the central letter stimuli and in active conditions they had to report occurrence of pre-defined targets in a rapid serial letter stream. Onset of the central letter stream enhanced activity in V1 representations of the stimulated region. Within representations of the periphery activation decreased and finally turned into deactivation with increasing distance from the stimulated location. This pattern was most pronounced in the active conditions and during the presence of peripheral stimuli. Active search for a target did not lead to additional enhancement at areas representing the attentional focus but to a stronger deactivation in the vicinity. Suppressed neuronal activity was also found in the non distracter condition suggesting a top-down attention driven effect. Our observations suggest that BOLD signal decreases in primary visual cortex are modulated by bottom-up sensory-driven factors such as the presence of distracters in the visual field as well as by top-down attentional processes.

## Introduction

Presenting visual stimuli at a certain location in the visual field increases neural firing in corresponding portions of primary visual cortex. Stimuli in adjacent visual field regions stimulate adjacent cortical regions, an observation reflected in the term retinotopy.

Retinotopic organization of the visual cortex can also be observed non-invasively in humans by means of fMRI [1], [2]. Here, BOLD signal increases relative to watching a blank screen (baseline condition) in those visual subareas that represent the stimulated visual field region. In other words, a positive BOLD signal seems to correlate with increased synaptic activity and neural firing [3], [4].

However, often enough the positive BOLD response is accompanied by negative signal changes (i.e., a reduction compared to baseline) in other areas of the visual system [5]–[7]. Until recently, such effects have been routinely discounted either because their origin was thought to be vascular and unrelated to neural firing rates (blood stealing) or because they were regarded as meaningless, non-specific and largely stimulus independent. This is surprising given the fact that experiments in laboratory animals have shown a long time ago that the presence of a visual stimulus does not only change the firing rate in its own cortical representation but also in adjacent visual cortex. Such stimulus-driven center-surround effects are considered an important mechanism in visual perception [8]. Hence, there are good reasons to believe that BOLD changes in visual cortex that is not directly stimulated reflect some meaningful aspect in visual processing.

In the present study, we therefore aimed at demonstrating that activity changes in visual cortex representing the surround of a stimulus indicate more than mere epiphenomena as blood steal and reflect crucial neural processes. To do so, we investigated whether negative BOLD changes are modulated independently from positive effects. Further we tested 1) whether these activity changes follow a center-surround or Mexican hat distribution and 2) if they depend on the composition of the visual scene and on top-down attentional control as a sign of their functional significance.

We measured activity changes induced by a foveally presented letter stream relative to a preceding fixation baseline. In order to assess surround effects, BOLD responses were not only measured in representations of the fovea but also in eight ring-shaped regions with increasing eccentricity from the fovea. The following four conditions were assessed. We compared an empty vs. a cluttered visual scene as surround effects are known to be influenced by the presence or absence of distracting stimuli [5]. Further, in order to account for attentional modulation we compared an active target search with passive watching in order to disentangle bottom-up and top-down driven surround effects.

## Results

### Behavioral Data

We assessed subjects' ability to discriminate targets from non-targets by calculating d′ from hit and false alarm rates (http://memory.psych.mun.ca/models/dprime/performance). In both distracter present and distracter absent trials d′ was larger than 2.4 indicating that subjects were very well able to discriminate targets from non-targets and were far from guessing. They adopted a rather conservative bias (C = 1.12) which is reflected by a very low false alarm rate (<1%) and low hit rates (54%, in the non-distracter condition, 53% in the distracter condition). A one-way repeated-measure ANOVA revealed no significant difference across these conditions, suggesting that subjects achieved sufficient suppression of the peripheral distracters.

### fMRI Data

**Figure 1:**
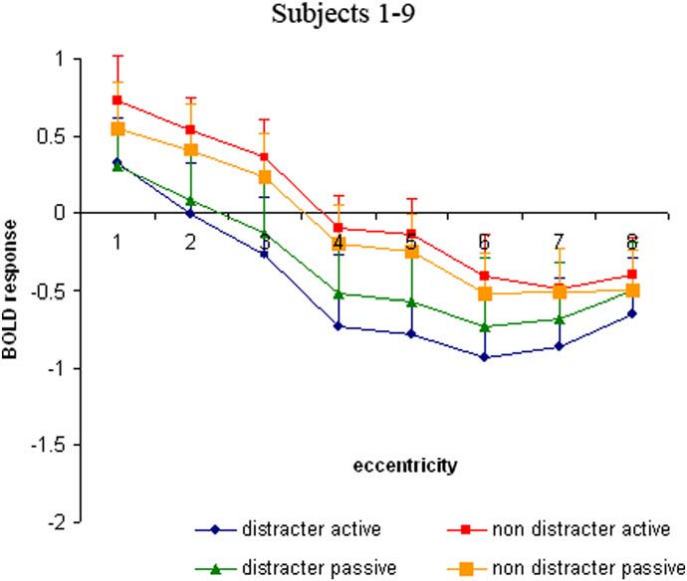
Activity distribution across the representations of ring 1 to 8. The BOLD responses were assessed in individual ROIs and then averaged across subjects.

Across all conditions we observed the strongest BOLD signal modulation in the region of visual cortex representing the innermost ring, i.e., the center of attention. With increasing distance from the center, BOLD signal in the respective Regions of Interest (ROIs) progressively decreased and eventually fell under the level of the baseline condition where subjects fixated the central cross (Fig. 1). Across all eccentricities, BOLD signal was lower in the distracter condition than in the non distracter version. The distracter condition revealed two further aspects: (i) higher BOLD signal values at the most remote eccentricity than in the more central adjacent region and (ii) a difference in BOLD signal between active and passive runs with lower values in the active condition. This effect was more pronounced in the right hemisphere.

These observations were confirmed by the statistical analysis: The three-way ANOVA showed significant main effects for the factors ‘scene’ (distracter vs. non distracter) [F (1, 8) = 36.62; p<0.001] and ‘eccentricity’ (ring 1–8) [F(2.65, 21.16) = 31.17; p<0.001]. No significant main effect was revealed for the factor ‘attention’ (active vs. passive) [F (1, 8) = 0.44; p = 0.52] but there was a significant interaction of ‘hemisphere’ and ‘attention’ [F(1, 8) = 6.72; p<0.03] and ‘attention’ and ‘scene’ [F(1, 8) = 21.38; p<0.002]. Post-hoc tests revealed that active vs. passive tasks differed only in the presence of distracters [F(1, 8) = 10.04; p<0.01] and that this effect was lateralized to the right hemisphere [F(1, 8) = 7.99; p<0.02].

A pairwise comparison between representations of the outermost ring 8 and the adjacent ring 7 revealed that in both active and passive runs of the distracter task the signal was less negative at ring 8 vs. ring 7 (−0.73 vs −0.95 %; t = −2.91, df = 8; p<0.02) corresponding to the ‘brim’ of the assumed Mexican hat. No such pattern could be observed in the non-distracter version (active condition: t = −1.05, df = 8; p = 0.33; passive condition: t = 0.09, df = 8; p = 0.93).

**Figure 2:**
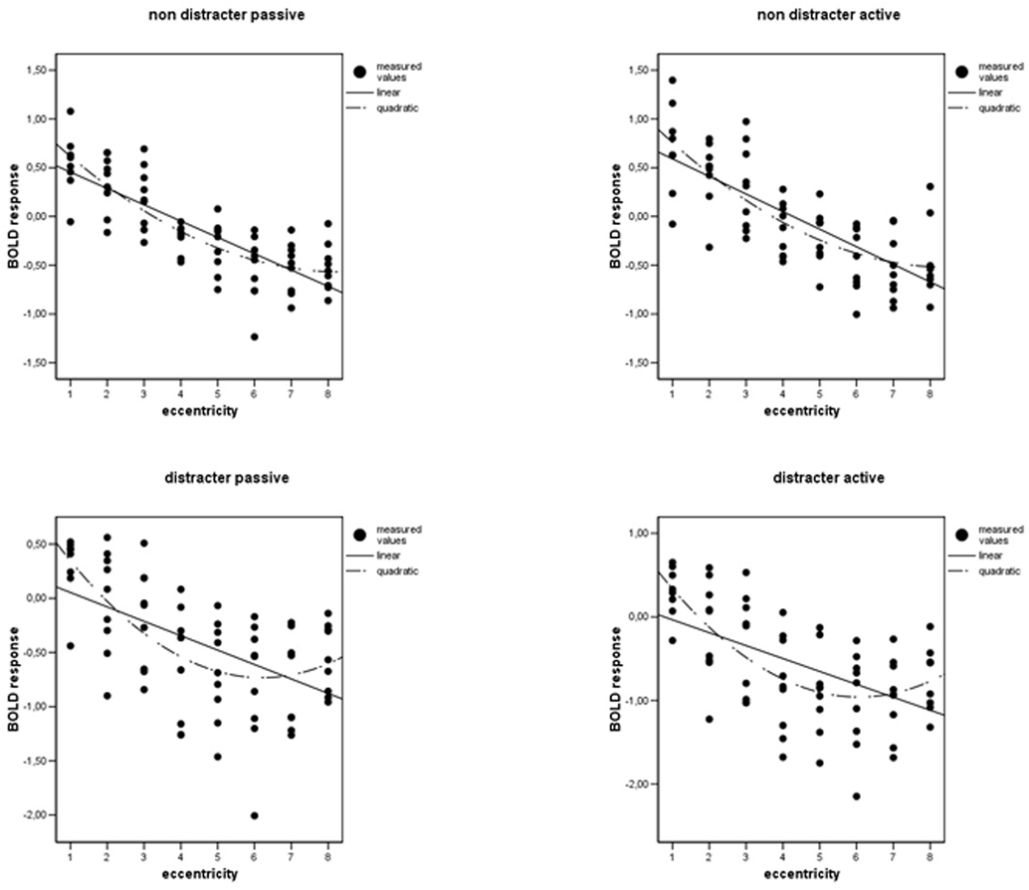
Regression based curve estimations for all four conditions.

To further investigate the notion of a Mexican hat distribution we calculated a regression based curve estimation. In the non distracter condition the linear function fitted the data nearly as well as the quadratic function (Fig. 2): quadratic function, active condition (F(2, 68) = 50.70, R^2^ = 0.60, p<0.0001; quadratic function, passive condition (F(2, 68) = 70.86, R^2^ = 0.68, p<0.0001), linear function, active condition (F(1, 69) = 88.92, R^2^ = 0.56, p<0.0001), linear function, passive condition (F(1, 69) = 118.64, R^2^ = 0.63, p<0.0001). Yet, in the distracter condition the quadratic function allowed for a clearly better data fit: quadratic function, active condition (F(2, 68) = 24.52, R^2^ = 0.42, p<0.0001), quadratic function, passive condition (F(2, 68) = 22.46, R^2^ = 0.40, p<0.0001), linear function, active condition (F(1, 69) = 28.69, R^2^ = 0.29, p<0.0001), linear function, passive condition F(1, 69) = 28.56, R^2^ = 0.29, p<0.0001).

As the additional parameter of the quadratic function allows for more flexibility, it could be that quadratic functions show a better fit by default. Therefore, we tested whether the difference is just due to the different number of parameters or whether it is large enough to justify the more complicated model. This was determined by computing the extra sum-of-squares F test where the null hypothesis assumes that the simpler model is correct. For every condition, this test rejected the null hypothesis in favour of the quadratic function but this effect was most significant for the distracter present conditions: (F(1,69) = 15.26, p<0.001 for the distracter active and F (1,69) = 12.56, p<0.001 for the distracter passive condition, (F(1,69) = 4.53, p<0.04 for the non distracter active condition and F(1,69) = 6.99, p<0.01 for the non distracter passive condition). These analyses show that a Mexican hat type of activation pattern mainly emerges when distracters are present.

### Between subjects variation

**Figure 3:**
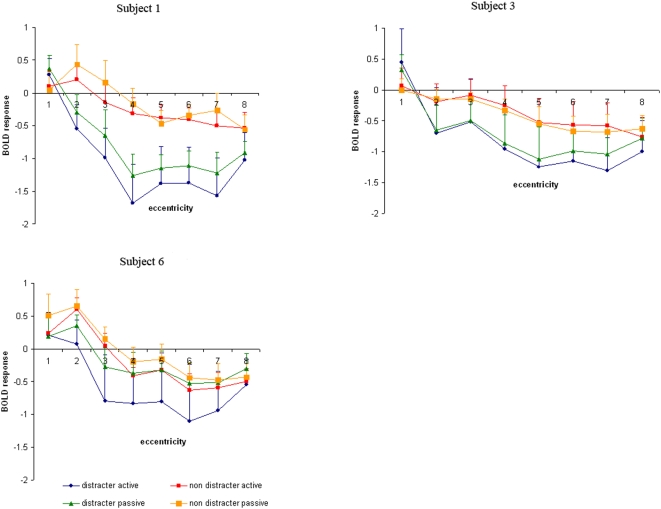
BOLD signal of three individuals.

Although the general pattern of a Mexican hat was present across all subjects, it should not be left unmentioned that the exact spatial pattern varied considerably across subjects (Fig. 3). For example, subject 1 showed an activity sink (or trough) at position 4 and subject 6 at position 6.

## Discussion

RSVP (rapid serial visual presentation) onset increased activity in representations of the fovea whereas a signal decrease was observed in surrounding primary visual cortex. This decrease correlated with distance, however, at the furthest representations the signal started to increase again. This pattern was observed across all tasks.

The reduced BOLD response (relative to baseline) in the periphery could not be attributed to putative hemodynamic epiphenomena as blood steal. If this was the case, the peripheral signal should be the more negative the more positive the foveal signal. This pattern, however, was not observed in our study: the signal decrease in the periphery was more pronounced in distracter present trials although the foveal signal was not higher in this condition. Moreover, active search for a target decreased activity in this condition further, but only in the periphery. Hence, while we cannot rule out that blood steal had some effect in our study, it certainly cannot account for all the effects observed.

What other explanation remains? The fact that the center-surround pattern was observed in both passive and active tasks suggests that this effect was bottom-up driven. This is further underpinned by the fact that the presence of distracters changed activity even in trials where subjects were not engaged in an active task. The latter observation points to lateral horizontal connections within V1 as a possible source of the observed signal variations. It has been shown that the response of a neuron to a stimulus within its classical receptive field can be strongly suppressed by another stimulus outside the neuron's receptive field. This has been attributed to lateral inhibition [9]–[14]. In our case, this would, however, only provide a good explanation for reduced responses to the foveal stimulus. The signal in the periphery should nevertheless be more pronounced than when no stimuli are present. Thus, attention offers the only conclusive explanation for all the observed effects. Attention can operate in a voluntary, top-down controlled mode but also in an automatic, bottom-up mode such as in attentional capture. Our RSVP stimulus like any sudden onset stimulus captured attention. Attention has been suggested to operate in a push-pull manner, explaining why the foveal increase was accompanied by signal decreases in the periphery. In accordance with previous research, this attention-driven center-surround modulation showed a Mexican hat distribution [15], [16].

On top of this exogenously driven modulation, however, voluntary attentional control during the active letter search attenuated activity only in the periphery and only when distracters were present. Many studies have shown that directing attention voluntarily to a peripheral region while keeping central fixation (covert attention) enhances activity in representations of the attended region across various visual areas [17], [18]. This observation has been attributed to the need to increase visual processing capacities which are usually lower in the periphery than at foveal representations. Consequently, attention-related enhancement has been found to be largest when hard-to-be perceived stimuli are presented in the periphery [19]. Here, on the other hand, we used easily perceivable stimuli presented to the fovea of our subjects. In such a setting attention exerts only little effect on the stimulated region and attention mainly serves to suppress peripheral distracters. This interpretation is supported by a deoxyglucose uptake study on behaving monkeys [20] showing a strong V1 suppression outside the attended area and only little or no enhanced activity at areas representing the attended area. The nature of this reduction of neuronal activity in regions representing the area outside an attended region could be clarified by a study with functional magnetic resonance imaging and electrophysiological recording [21]. It could be shown that a significant component of negative BOLD response originates in a decrease of neuronal activity. This finding argues against blood stealing and supports the idea of attentional suppression.

If attenuated BOLD responses simply reflected a push-pull mechanism or blood steal, then they should not occur without an enhancement at some other brain region. If, on the other hand, negative BOLD reflected true attentional suppression of peripheral distracters than it should be observable without a positive effect on foveal representations. The latter is exactly what we found. Hence, this observation clearly goes beyond a simple pull-push mechanism. Rather, at least with the presence of highly salient foveal stimuli, top-down attention mainly serves to suppress distracting information in the periphery.

## Materials and Methods

### Subjects

Nine healthy right-handed subjects (6 females, mean age 25 years, range 22–31) participated in the study. All subjects had normal color vision. Ametropia was corrected with glasses mounted into the goggles used for presentation. All subjects signed an informed consent form and were moderately financially rewarded for their participation in the study conducted in conformity with the declaration of Helsinki and approved by the local ethics committee.

### Regions of interest (ROIs) and meridian mapping

Regions of interest(s) (ROI) in V1 were defined in separate runs before the actual attention experiment started. To do so, we first defined the borders of the visual areas by mapping the horizontal and vertical meridians [22]. Each subject completed one 6-minute run, which included 8×20 s stimulation of either the horizontal or the vertical meridian with piece of cake like checkerboard stimuli reversing at 8 Hz, and 3×20 s fixation periods at the beginning, in the middle and the end of each run.

**Figure 4:**
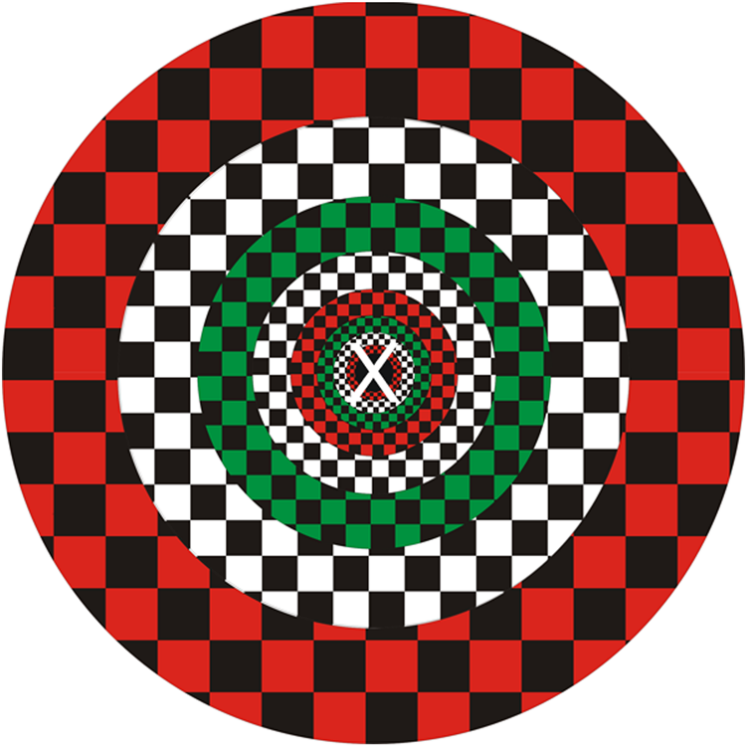
Rings that were used to map visual field regions with increasing eccentricity. Note, that the rings were presented sequentially and that each ring was presented with changing colors. In this figure the position of the stimulus letters used in the task is shown. The outer parts of the letter end in the second ring. See table 1 for exact data of the rings.

In a second step, ROIs were defined as subareas of V1 representing circular regions of increasing eccentricity from the fixation center. To map these regions we used checkerboard rings of eight different eccentricities, which flickered at 8 Hz in the colors black-white, black-red and black-green (Fig. 4). The two innermost rings fell within the area where the central task was presented later in the experiment. We created our stimuli by accounting for the cortical magnification factor in order to activate cortical regions of similar size [23], [24]. Hence, the ring at the central position was thinnest while the most peripheral one was the broadest (see table 1 for exact data). Rings were presented in four blocks within a 12-minute session. In each block every ring was presented for 20 s. The sequence of rings within a block was pseudo-randomized avoiding that rings of adjacent eccentricities were presented one after the other. The four blocks were separated by 20 s fixation periods.

**Table 1:** Inner and outer radius of the presented rings

ring	inner radius	outer radius
	[visual angle]	[visual angle]
1	0.38°	0.56°
2	0.56°	0.71°
3	0.75°	1.2°
4	1.21°	1.78°
5	1.78°	2.81°
6	2.9°	4.5°
7	4.5°	6.94°
8	7.03°	10.78°

### Visual attention paradigm

Subjects fixated streams of letters in a RSVP mode. Single white letters (2.25° height) were presented at the center of the screen against a black background. In order to create a sufficiently demanding task, each letter was presented only for 120 ms with no gap between letters. Subjects' task in the active condition was to press a button whenever they detected the letters ‘O’ or ‘X’ while ignoring the non-target letters A, B, C, E, F, G, H, K, L, N, Q, R, S and T. On average 121 targets were presented among 947 non-target letters, i.e. target frequency was 11%. BOLD responses in active trials were compared to those elicited in passive trials where subjects merely watched the letter stream passively while maintaining central fixation. Although it is always hard to control what subjects actually do during passive tasks we nevertheless preferred this task over an active control task in which subjects direct attention elsewhere for the following reasons: An active control task in the visual domain where attention is directed for example to a peripheral location would only allow us to contrast two maps of differentially distributed spatial attention occluding all effects common to both tasks. An active task in which attention is directed to another modality instead would not be a good control either as it has been shown that attention to auditory stimuli alters activity in visual cortex [25], [26]. Therefore, a passive control task seemed the lesser evil.

**Figure 5:**
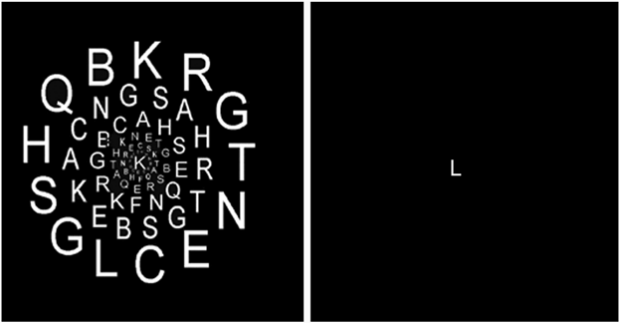
Examples for the two different visual scenes employed. Left: central letter stream, no distracters. Right: central letter stream plus distracter letters.

There were two versions of the task: In the non-distracter task, the central letters were presented on an otherwise empty black screen. In the distracter version a cluster of letters surrounded the central RSVP (Fig. 5). Six rings of letters were displayed which corresponded in size and eccentricity to the six peripheral rings used for ROI mapping. Each ‘distracter ring’ contained 12 to 13 letters and there were 77 distracter letters altogether. The distracters were presented continuously throughout an entire run.

Subjects performed two 8-minute experimental runs, one consisting of the non-distracter version, the other of the distracter version of the task (in counter-balanced order across subjects). Rather than changing conditions block-by-block within a run, this procedure avoided strong changes of the BOLD signal that would have been induced by switching the distracters off and on [27]. Each run contained four active and four passive blocks of 40 s duration each, presented in randomized order. Between blocks a white fixation cross was presented in the central circle for 15 s which then changed its color either to red or green for another 5 s. A red cross indicated that a passive, a green cross that an active task would follow. Subjects completed four training blocks outside the scanner.

### fMRI procedures

fMRI data were acquired with a 3T MRI system (Allegra, Siemens, Erlangen, Germany). Stimuli were presented by the ERTS software package (BeriSoft, Cooperation, Frankfurt, Germany). They were displayed through MR-compatible goggles (Resonance Technology) simulating a 30×20° visual field. Functional images were obtained by using a gradient echo echoplanar imaging sequence (TR = 2000 ms; TE = 62 ms, 32 slices, 3 mm thickness, gap 0.1 mm, in plane resolution 3 mm×3 mm). High resolution (1×1×1 mm^3^) structural images were acquired of each subject using a T1-weighted sagittal MPRage sequence.

For the attention experiment we collected 482 functional volumes, for the ROI localizer 371 volumes and for the meridian mapping 191 volumes.

### Data analysis

fMRI Data were analyzed with Brainvoyager QX software (BrainInnovation, Maastricht, The Netherlands). Functional data were preprocessed including motion correction, high pass filtering (3 cycles per time course) and temporal smoothing (5 s FWHM). No spatial smoothing was applied. For further analyses anatomic data were transformed into stereotactic space [28]. Then functional data were co-registered with the 3D structural data sets to generate volume-time-courses that could be averaged per condition.

**Figure 6:**
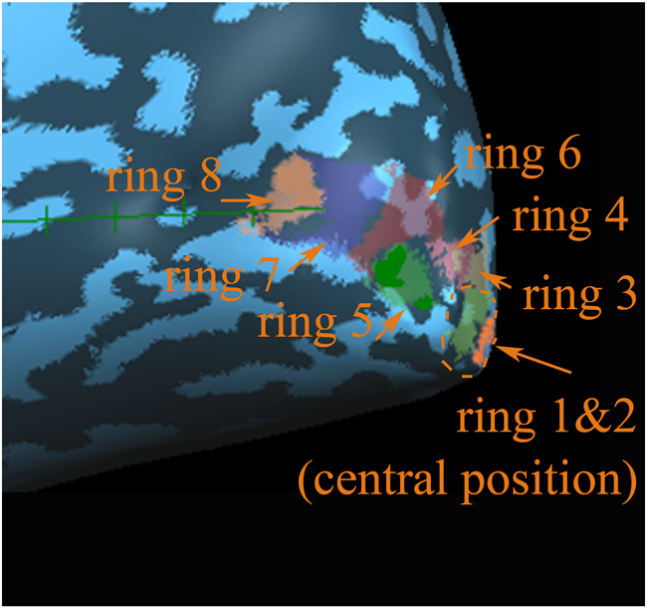
Mapping of the ring representations in primary visual cortex in one representative subject. Ring 1: innermost ring, ring 8: outermost ring.

For the definition of ROIs on the anatomical data set, white matter was segmented from gray matter for each hemisphere. Then the surface was reconstructed and inflated following the standard procedure implemented in the Brainvoyager software. On this surface the region activated by the horizontal meridian along the calcarine sulcus and delimited ventrally and dorsally by representations of the vertical meridian was defined as V1. With the data from the ring mapping experiment, a fixed-effects general linear model was calculated with each ring serving as a predictor. Then contrasts between the ring of interest and all other rings were calculated. For this procedure we had to use varying but never weaker than <0.05 significance levels to assure that the activated cortical regions neither overlapped nor had large gaps between them. The latter would have been inevitably the case had we applied a fixed threshold. According to their eccentricity the presented rings activated visual cortical areas varying from occipital pole to more anterior regions. Finally, from the total cortical surface activated by each ring the portion corresponding to pre-defined V1 was selected (Fig. 6).

For each ROI, the BOLD response to the RSVP in each condition was averaged from the volume-time-courses covering the 20 volumes recorded after onset of the RSVP. The fixation period preceding the RSVP task served as a baseline. The BOLD response was averaged across hemispheres and then entered into a repeated-measure ANOVA for group analysis. The ANOVA included the factors ‘attention’ (active vs. passive), ‘scene’ (non-distracters vs. distracters) ‘eccentricity’ (ring 1–8) and ‘hemisphere’. Post-hoc tests were computed whenever significant interactions effects had occurred in the main analysis. All values were Huynh-Feldt corrected.
